# Comprehensive analysis of the GATA transcription factor gene family in breast carcinoma using gene microarrays, online databases and integrated bioinformatics

**DOI:** 10.1038/s41598-019-40811-3

**Published:** 2019-03-14

**Authors:** Shan Yu, Xuepeng Jiang, Juan Li, Chao Li, Mian Guo, Fei Ye, Maomao Zhang, Yufei Jiao, Baoliang Guo

**Affiliations:** 1 Department of Pathology, the Second Affiliated Hospital of Harbin Medical University, Harbin, 150001 China; 2 Department of General Surgery, the Heilongjiang Power Hospital, Harbin, 150090 China; 3 Department of Orthopedics, the Second Affiliated Hospital of Harbin Medical University, Harbin, 150001 China; 4 Department of Neurosurgery, the Second Affiliated Hospital of Harbin Medical University, Harbin, 150001 China; 5 Department of Pathology, Harbin Medical University, Harbin, 150001 China; 6 The Key Laboratory of Myocardial Ischemia, Department of Cardiology, the Second Affiliated Hospital of Harbin Medical University, Harbin, 150001 China; 7 Department of General Surgery, the Second Affiliated Hospital of Harbin Medical University, Harbin, 150001 China

## Abstract

Integrated studies of accumulated data can be performed to obtain more reliable information and more feasible measures for investigating the potential diagnostic and prognostic biomarkers of breast cancer and exploring related molecular mechanisms. Our study aimed to explore the GATA family members involved in breast cancer by integrating data from The Cancer Genome Atlas (TCGA), Gene Expression Omnibus (GEO) and other online databases. We performed an integrated analysis of published studies from GEO and analyzed clinical data from TCGA and GTEx to evaluate the clinical significance and prognosis values of the GATA family in breast cancer. GATA3 was found to be upregulated and exhibited a favorable value in the diagnosis and prognosis of breast cancer. Through this study, we identified possible GATA3-correlated genes and core pathways that play an important role, which requires further investigation in breast cancer.

## Introduction

GATA transcription factors are defined as a family of transcription factors characterized by their DNA-binding specificities to the “GATA” DNA sequence^1^. These transcription factors are highly conserved by amino acid sequence identity within the binding target site, conforming to the consensus motif WGATAR (where W = A or T and R = A or G)^2^. Currently, six members are in the GATA transcription factor family, named in order of when they were discovered, from “GATA1” to GATA6”.

GATA1 and GATA2 were first found to be involved in regulating the cell cycle and in proliferation during primitive hematopoietic development^3^. More recent studies have revealed that GATA1 and GATA2 also participate in the progression of breast cancer and prostate cancer via EMT processes^4,5^. GATA3 plays an integral role in luminal cell differentiation in mammary glands^6^. Consequently, GATA3 has recently been drawing scientists’ attention in breast cancer, but the supporting evidence is inconclusive^7^. In addition, the prognostic significance of GATA3 in breast cancer and other malignancies remains controversial according to differing research results^8^. GATA4, GATA5, and GATA6 were also classified as endodermal GATA factors. The altered expression of GATA4, GATA5, and GATA6 is associated with various malignancies broadly from the gastric tract, lungs, ovaries, and even the brain^9^. However, their role in cancer as an oncogene or a tumor suppressor gene is still uncertain.

Progressively, using gene regulation networks from large databases to develop an understanding of transcription factor functions has been widely accepted by the biology research field. Thus far, our current state of knowledge about GATA factors in the context of human cancers is still limited. Despite the distinctive role of individual GATA members in the development and progression of human cancers, the integrated functions and prognostic values of different GATA members in breast cancer are largely unexplored.

The present study aimed to systemically investigate the expression and prognostic values of GATA family members with potential gene functions in breast cancer by using integrated large databases. We explored the characterization of the GATA family member gene status of breast cancer patients from expression patterns to prognostic values and potential clinical pathology application to provide a comprehensive understanding of GATA family utilities in breast cancer.

## Methods

### GATA family Expression Data Pan-cancer analysis

To analyze the expression of the GATA family gene in a variety of malignancies, the TCGA, GTEx and Oncomine online databases were accessed for the visualization of gene expression. Oncomine is an online cancer microarray database used to facilitate and promote discoveries from genome-wide expression analyses. The pan-cancer studies in Oncomine were selected to compare the expression levels in tumors vs normal tissues. The selection criteria for the Oncomine studies were P < 0.05 as a threshold, 2-fold change and gene rank in the top 10%. The P-values, fold changes, and cancer subtypes were extracted. In addition, we compared GATA family mRNA RNA-Seq data from clinical specimens of pan-cancer including breast cancer tissue versus normal tissue from the data imported from TCGA and GTEx by Gene Expression Profiling Interactive Analysis (GEPIA)^10^. Moreover, the expression profile of the GATA family members in each breast cancer subtype from TCGA was visualized by Oncomine in a log2 median-centered ratio.

### Breast Cancer Gene-Expression Miner v4.1 with survival meta-analysis

To analyze the association among the expression levels of each member of the GATA family and the clinicopathological features of breast cancer, the online database Breast Cancer Gene-Expression Miner v4.1 (BC-GEM, bcGenExMiner v4.1), which comprises 36 genomic datasets, was used (http://bcgenex.centregauducheau.fr/BC-GEM/GEM-requete.php)^11,12^. All validated GATA family members in the 36 datasets containing over 3000 cases from BC-GEM were pooled for a survival meta-analysis.

### GOBO expression analysis

The mRNA expression levels of GATA3 in different clinical parameters were analyzed by the GOBO database v 1.0.2 (http://co.bmc.lu.se/gobo/gsa.pl). GOBO is an accessible tool that contains 1881 breast cancer tumor sample datasets, including clinical characteristics^13^. The GEO datasets GSE11121, GSE7390, GSE2034, GSE5327, GSE2603, GSE3494, GSE1456, GSE6532, GSE4922 and GSE12093 were combined to perform an analysis on the GATA3 expression differences within the breast cancer molecular subtypes, PAM50 subtypes, ER status and tumor grade. Additionally, the expression in the breast cancer stage was compared by one-way ANOVA.

### The relapse-free survival Kaplan-Meier analysis

To analyze the prognostic values of GATA3 in breast cancer samples with different clinical conditions, the Kaplan-Meier plotter for breast cancer (www.kmplot.com)^14^ was used to display relapse-free survival (RFS) with different clinical parameters separated by median expression with the probe 209602_s_at. The log-rank P-value was calculated with <0.05 considered statistically significant.

### Immunohistochemistry expression pattern

The Human Protein Atlas (HPA) is an open access program that maps all human proteins in cells and tissue samples (https://www.proteinatlas.org/)^15^. IHC data for clinical potential application were extracted from the Human Protein Atlas in both normal breast tissue and breast cancer. A high and low expression pattern was selected to validate the potential applicants for GATA3 in breast cancer prognosis prediction.

### Gene expression omnibus data mining and identification of DEGs

Microarray profiles related to breast cancer were downloaded from the GEO database (http://www.ncbi.nlm.nih.gov/geo/)^16^. For the GATA3 overexpression profile in breast cancer, the GSE24249 dataset was included in our analysis. GSE24249 is an expression profile with overexpressed GATA3 in the MDA-MB231 breast cancer cell line in a GPL570 Affymetrix Human Genome U133 Plus 2.0 Array platform^17^. The raw microarray data were extracted and normalized. R software was used with the limma package to identify the differentially expressed genes (DEGs). A volcano plot was applied to show the DEGs in GSE24249. Additionally, numerous DEGs were plotted to present the top upregulated and downregulated differential genes.

### Integrative bioinformatics analysis of the selected GATA family members

The achieved DEGs were pooled for Gene Ontology (GO) terms (biological process) using the GO Annotation database (released 2018-09-06) through the Gene Ontology Consortium (http://pantherdb.org)^18^, an online database that provides a comprehensive set of functional annotation tools for the interpretation of summed genes. The gene ratio and enrichment score with an FDR were calculated and plotted by R software with a GO enrichment^19^.

Kyoto encyclopedia of genes and genomes (KEGG) pathway enrichment analysis was performed by using the DAVID database (Version 6.7)^20,21^. All enriched pathways were plotted by using R software. FDR < 0.05 was set as a cutoff for significance.

The Search Tool for the Retrieval of Interacting Genes (STRING) database (version 10.5; http://string-db.org/)^22^, is used to explore protein-protein interaction information. To evaluate the interactive associations among overexpressed GATA-3-related DEGs, the interaction with a combined score >0.4 was selected and mapped. Then, the DEGs PPI network was constructed and visualized using Cytoscape software (version 3.5.1; www.cytoscape.org)^23^.

Gene set enrichment analysis (GSEA) was performed using the Molecular Signatures Database (MSigDB) software^24,25^. The overexpressed GATA3 group in the GSE24249 dataset was set as phenotype 1, and the vector group was set as phenotype 2. The goal of GSEA in our research was to identify the distribution of biological functions within the DEGs. An enrichment score (ES) was calculated. The gene ranking metric in the weighted ES was the 2-sided SNR, and the p-values were calculated using 1,000 permutations of the phenotype.

## Results

### Differential expression studies of the GATA family transcript in pan-cancer

**Figure 1:**
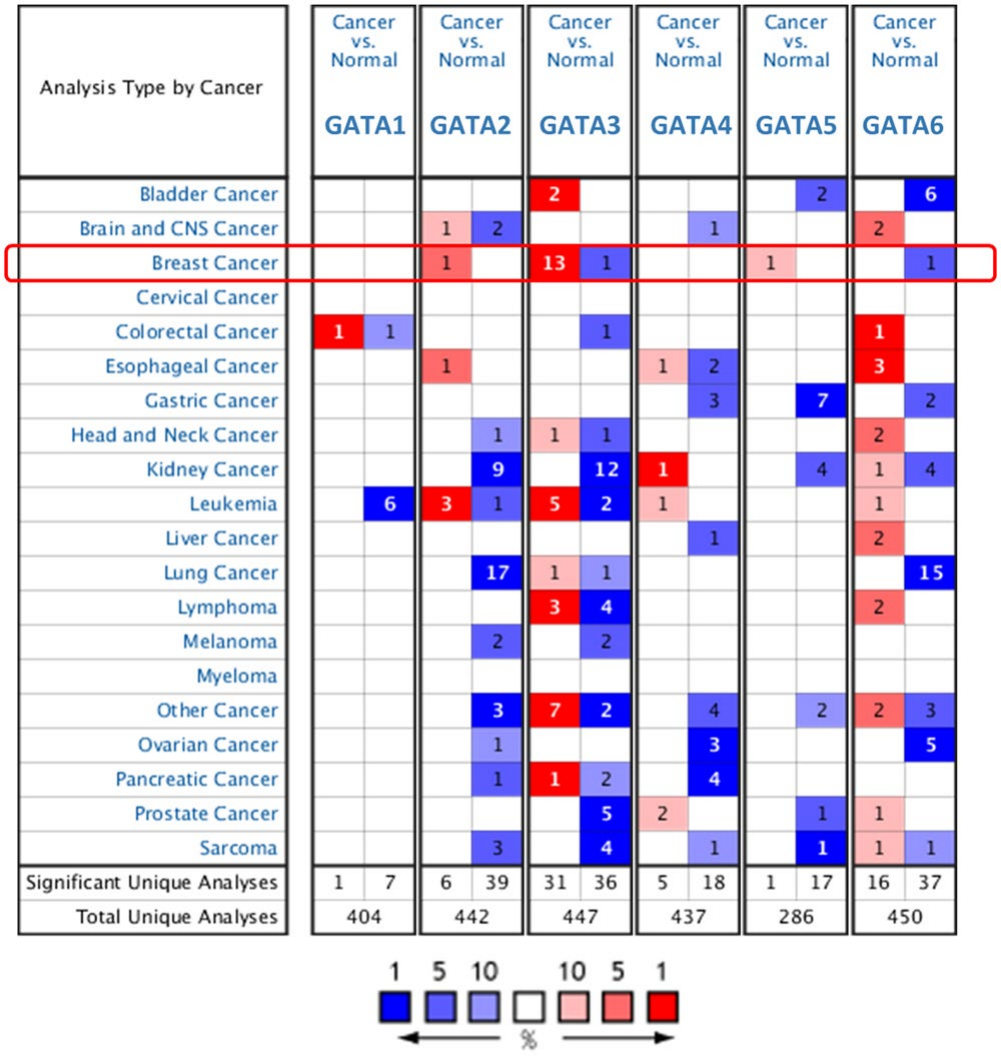
The mRNA expression levels of the GATA family genes according to the Oncomine database. The mRNA expression of the GATA family members (cancer vs normal tissue) in pan-cancers analyzed with the Oncomine database. The graphic demonstrates the numbers of datasets with statistical significance. Red: upregulation; blue: downregulation. The number in each cell represents the datasets that meet our threshold in each cancer type. Cell color was defined as the gene rank percentile in the study.

**Figure 2:**
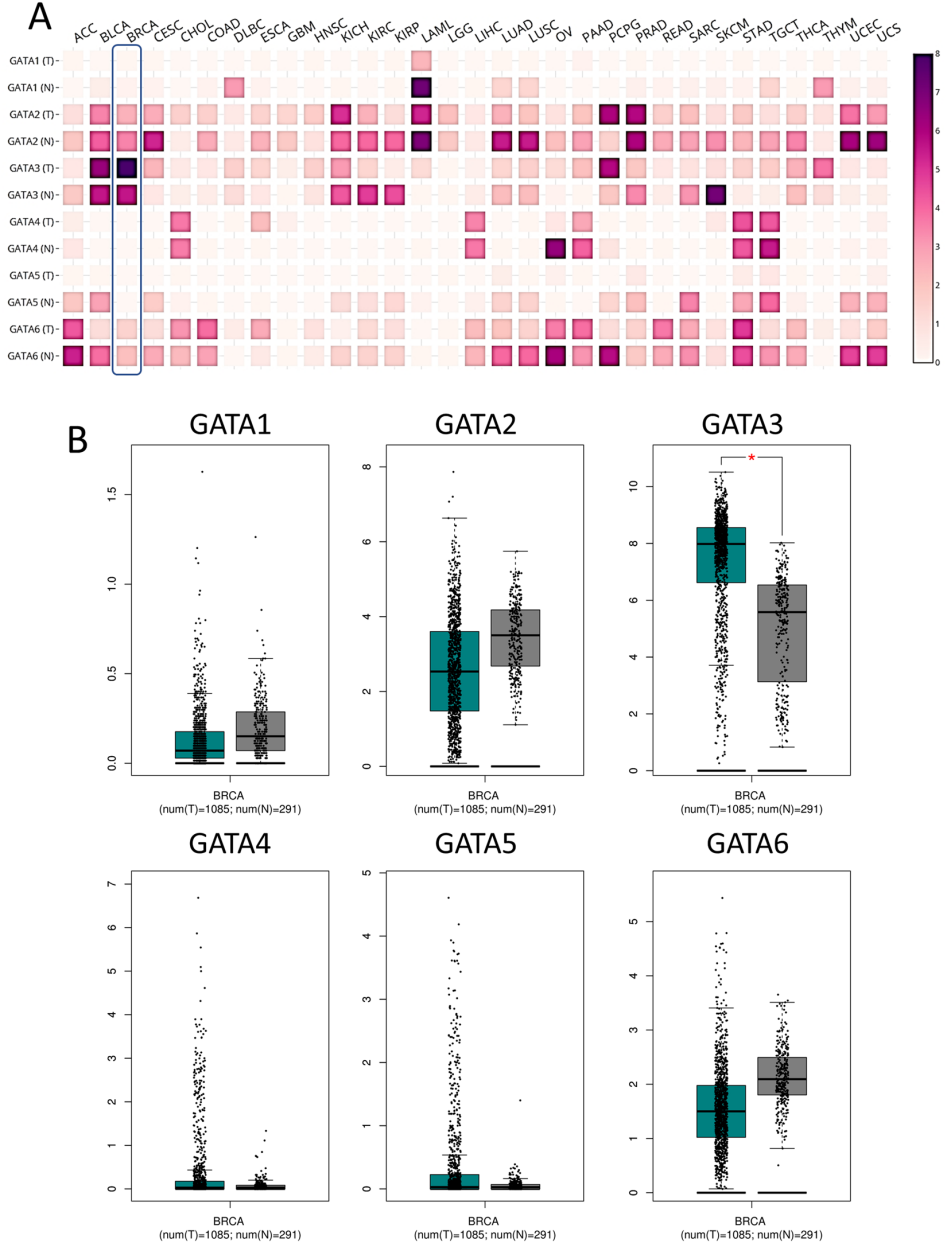
The RNA-Seq profile of the GATA family members in the pan-cancer analysis. (**A**) The heatmap indicates the expression after normalization by log2(TPM + 1) for log-scale compared with tumor and normal tissues in pan-cancer. The cancer abbreviation names are shown according to TCGA study abbreviations. (**B**) Boxplot of the expression profile of the GATA family members in breast cancer. A t-test was used to compare the expression difference between tumor and normal tissues.

The six GATA family members from GATA1 to GATA6 were explored in human cancers by the Oncomine online database. The top mRNA differences between cancer and normal tissues was analyzed by our selective criteria. As shown in Fig. 1, the Oncomine database contained a total of 404, 442, 447, 437, 286 and 450 unique studies involving the genes with GATA1 to GATA6, respectively. Interestingly, all GATA family members besides GATA3 were mostly downregulated in most kinds of cancers, with upregulated vs downregulated study numbers (GATA1 1:7; GATA2 6:39; GATA3 31:36; GATA4 5:18; GATA5 1:17; GATA6 16:37). In detail, the GATA3 mRNA expression level increased in 13 cases compared with one decrease in breast cancer. However, the other members did not show any significant validated study differences in breast cancer. In kidney cancer, GATA2, GATA3, GATA5 and GATA6 were mostly downregulated. In lung cancer, GATA2 and GATA6 were significantly downregulated genes in 17 and 15 studies, respectively. The RNA-Seq expression heatmap (Fig. 2A) showed that GATA3 was significantly increased in breast cancer tissues compared with normal tissues. Additionally, GATA3 expression in breast cancer was relatively higher than in any other normal tissue or cancer type in the pan-cancer profile.

### GATA family expression in breast cancer

**Figure 3:**
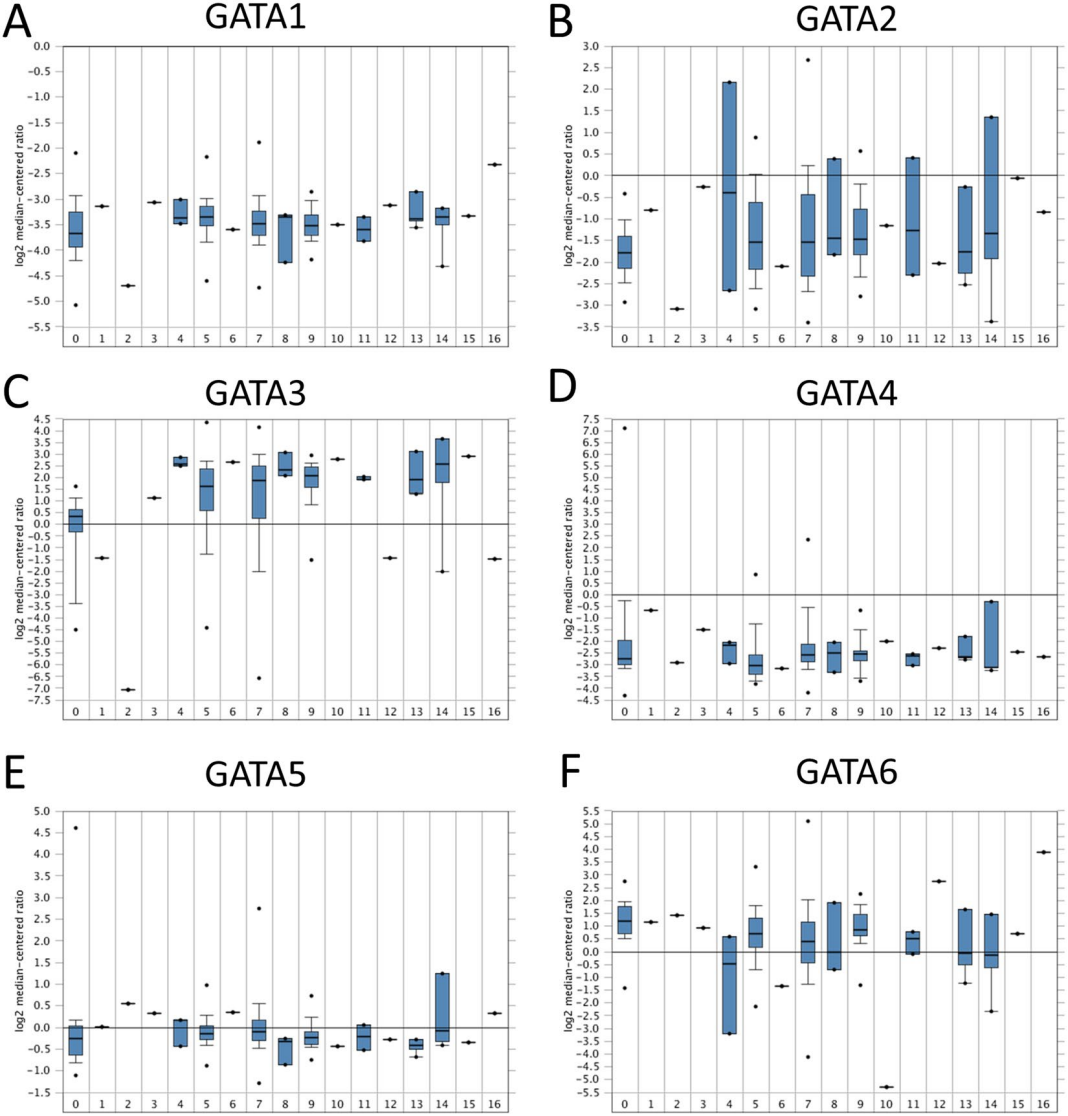
Box plots representing the mRNA expression levels of the GATA family genes in different types of breast cancer and normal controls in the TCGA database. 0 = Normal (61); 1 = Apocrine Breast Carcinoma (1); 2 = Breast Large Cell Neuroendocrine Carcinoma (1); 3 = Ductal Breast Carcinoma (1); 4 = Intraductal Cribriform Breast Adenocarcinoma (3); 5 = Invasive Breast Carcinoma (76); 6 = Invasive Cribriform Breast Carcinoma (1); 7 = Invasive Ductal Breast Carcinoma (392); 8 = Invasive Ductal and Lobular Carcinoma (3); 9 = Invasive Lobular Breast Carcinoma (36); 10 = Invasive Papillary Breast Carcinoma (1); 11 = Male Breast Carcinoma (3); 12 = Metaplastic Breast Carcinoma (1); 13 = Mixed Lobular and Ductal Breast Carcinoma (7); 14 = Mucinous Breast Carcinoma (4); 15 = Papillary Breast Carcinoma (1); and 16 = Pleomorphic Breast Carcinoma (1).

The boxplot of the RNA-Seq expression in 1085 breast cancer tissues vs 291 normal breast tissues demonstrated that GATA3 was significantly increased (Fig. 2B). For other GATA family members, no statistical significance was revealed in cancer tissues compared to normal tissues. These data indicated that the expression of GATA3 may have a major effect on breast cancers. In addition, we addressed the expression level of breast cancer subtypes from the TCGA database. Our results are also consistent with the RNA-Seq data in breast cancer in which, except for GATA3, all other GATA family members showed no significant difference compared with normal tissue control. GATA3 decreased only in apocrine, large cell neuroendocrine, metaplastic and pleomorphic breast cancer subtypes (Fig. 3C). These subtypes are known to have poor clinical outcomes, which indicates that GATA3 expression may be greatly involved in breast tumor malignancy. However, the case number of each rare subtype in the TCGA database is a limitation for accuracy estimation.

### The GATA family prognosis analysis with a combined dataset

**Figure 4:**
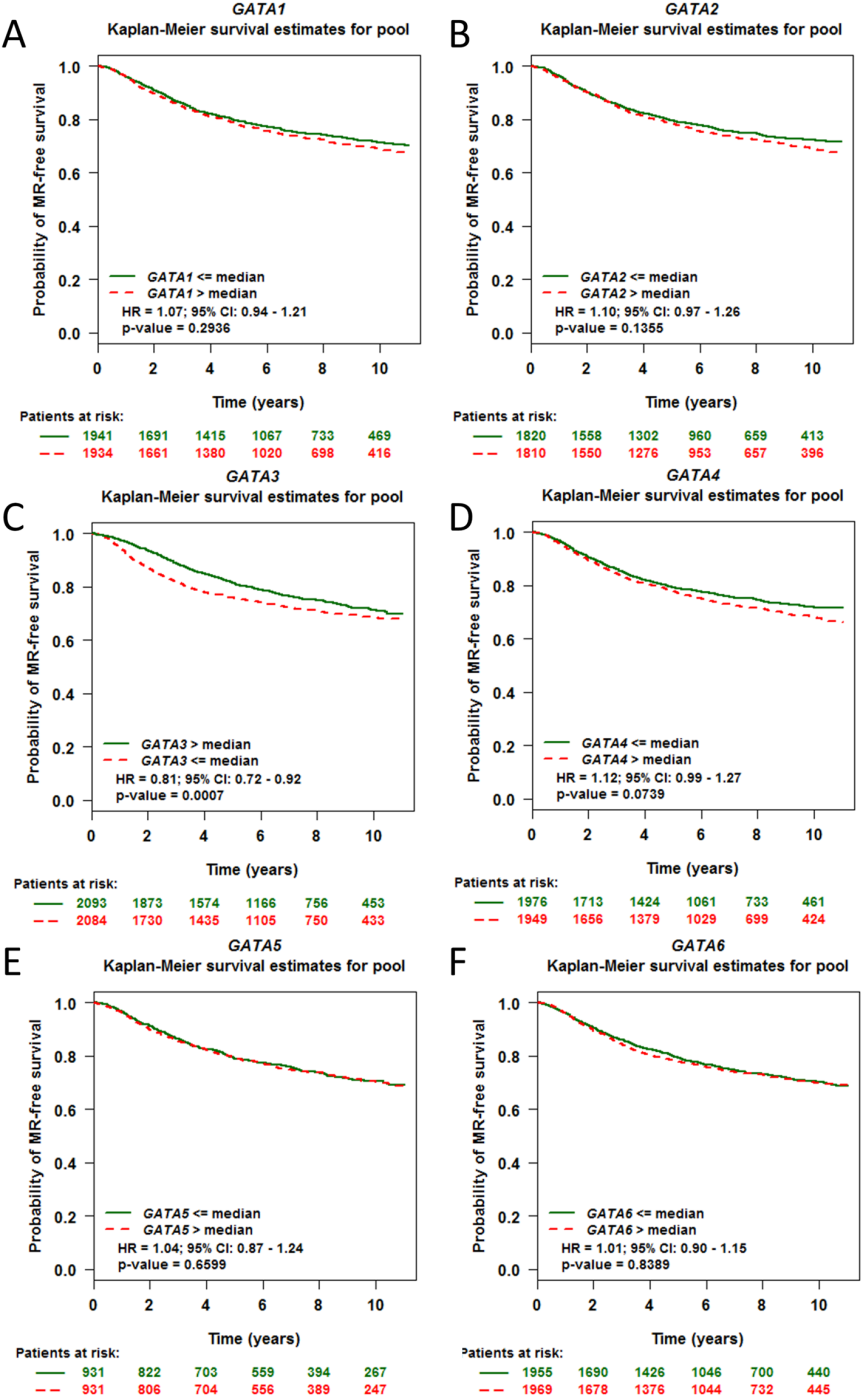
The prognostic values of the GATA family members in breast cancer. Metastatic Relapse (MR) is selected as a terminal event for the pooled Kaplan-Meier survival analysis. The median expression is set as a separate line for each GATA family member. A p value < 0.05 was considered statistically significant.

To date, little is known about the GATA family expression levels or their possible prognostic value, except for GATA3, in breast cancer. Therefore, we performed Kaplan-Meier survival analysis according to the median mRNA expression of GATA family members systematically by using bcGenExMiner v4.1. For GATA1, the median expression level for the pooled survival analysis could not be considered a significant marker for metastatic relapse-free survival with a total of 3875 breast cancer patients (HR = 1.07; 95% CI: 0.94–1.21, p-value = 0.2936, Fig. 4A). The median expression level of GATA2 (patients = 3630, HR = 1.10; 95% CI: 0.97–1.26, p-value = 0.1355, Fig. 4B), GATA4 (patients = 3952, HR = 1.12; 95% CI: 0.99–1.27, p-value = 0.0739, Fig. 4D), GATA5 (patients = 1862, HR = 1.14; 95% CI: 0.87–1.24, p-value = 0.6599, Fig. 4E) and GATA6 (patients = 3924, HR = 1.01; 95% CI: 0.90–1.15, p-value = 0.8389, Fig. 4F) also demonstrate the same negative results as GATA1. However, only GATA3 showed a positive effect in the survival analysis (patients = 4177, HR = 0.81; 95% CI: 0.72–0.92, p-value = 0.0007, Fig. 4C). The higher expression of GATA3 indicated a better survival time. A prognostic meta-analysis of the GATA family member results is shown in Fig. S1.

### The differential expression of GATA3 in breast cancer molecular subtypes, PAM50 subtypes, ER status, tumor stage and grade

**Figure 5:**
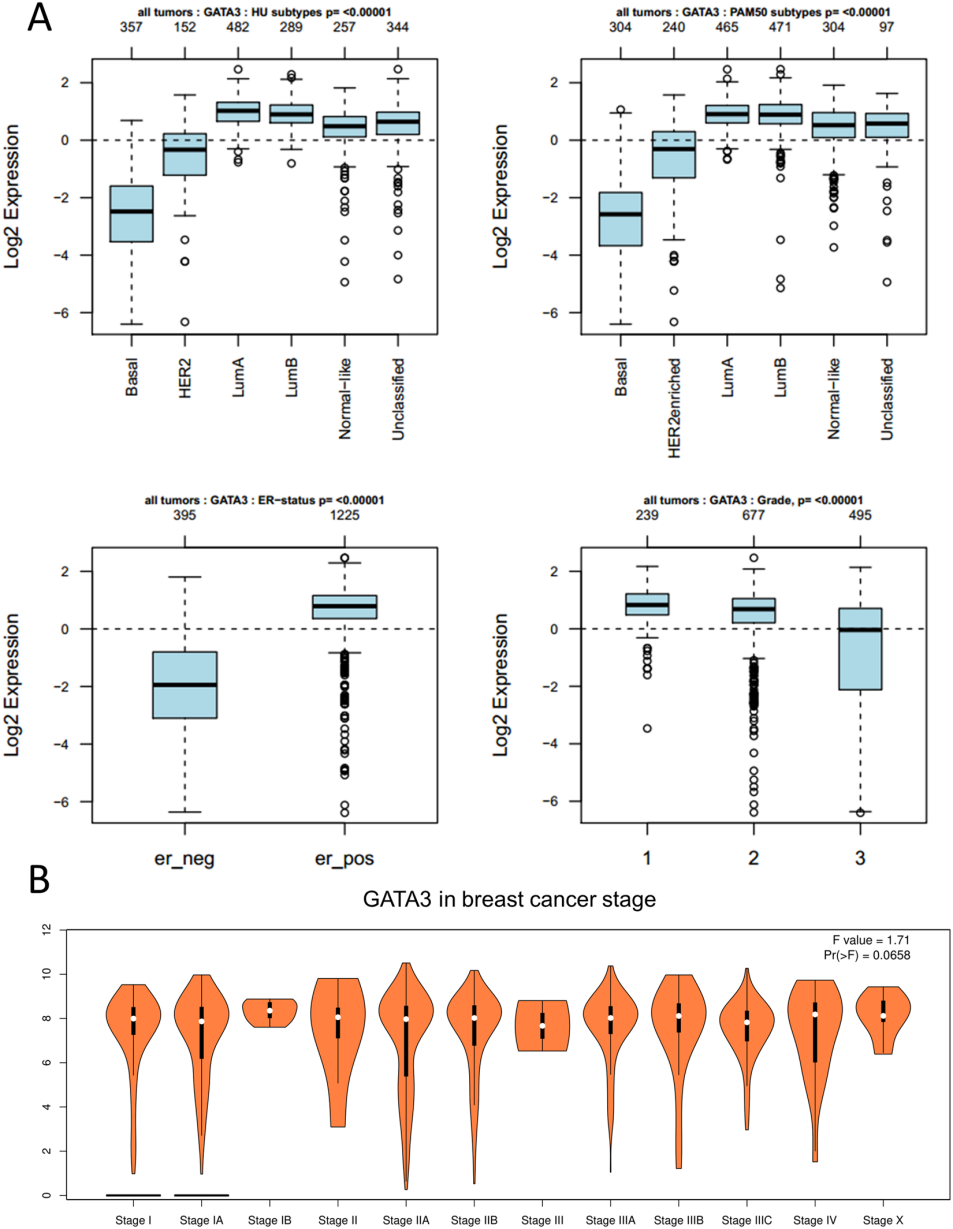
The relationship between the gene expression level of GATA3 in breast cancer subtypes, tumor grade and stages. (**A**) GATA3 expression in molecular subtypes; PAM50 classification; estrogen receptor and tumor grade. (**B**) GATA3 expression in breast cancer stages.

In breast cancer, only GATA3 could be considered a validated prognosis marker compared with the other GATA family members according to our results. Therefore, we further focused on the expression of GATA3 in different clinical parameters (Fig. 5A). Our results showed that GATA3 expression is lower in basal subtypes (n = 357) and higher in the luminal A subtype (n = 482). This expression difference is significant among the current molecular subtype classifications. For the PAM50 subtype analysis^26^, which is known to be a gene expression classifier of the intrinsic subtypes of breast cancer to assess both the prognostic and predictive values of adjuvant hormonal therapy in a study population of premenopausal women, we obtained the same results as the molecular subtypes. An ER-positive estrogen receptor status demonstrated a higher expression level of GATA3 (n = 1225). Interestingly, in tumor grade, grade 3 tumors showed a relatively lower expression compared with grade 2 and grade 1 tumors. However, there was no significant difference in GATA3 expression among clinical stages (Fig. 5B).

### Increased GATA3 expression levels are significantly associated with longer RFS times in PR-positive breast cancer and lymph node metastasis

**Figure 6:**
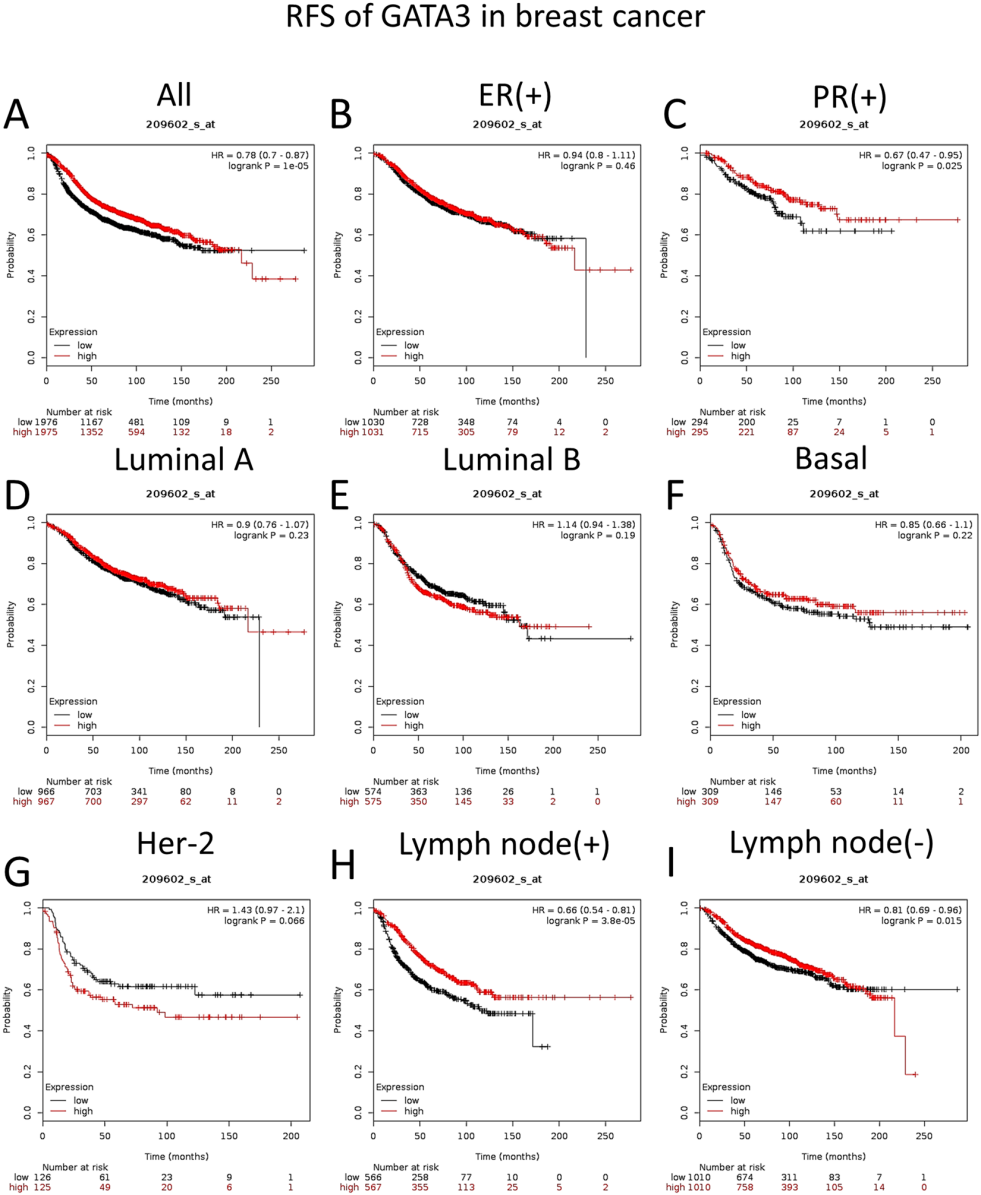
The prognostic values of GATA3 in breast cancer with clinical parameters from the KM plot. The median expression is set as the separation point. Red = High, Black = Low. (**A**) Pooled, all breast cancer (n = 3951). (**B**) Estrogen receptor-positive breast cancer (n = 2061). (**C**) Progesterone receptor-positive breast cancer (n = 589). (**D**) Luminal A subtype (n = 1933). (**E**) Luminal B subtype (n = 1149). (**F**) Basal subtype (n = 618). (**G**) Her-2 subtype (n = 251). (**H**) Lymph node positive status (n = 1133). (**I**) Negative lymph node status (n = 2020).

Our Kaplan-Meier analysis by KM-plotter showed that GATA3 expression is positively related to the RFS time if no clinical parameters are selected (Fig. 6A), which is consistent with our bcGenExMiner results. However, after we investigated the details of the patient group, we found that although GATA3 was higher in ER-positive breast cancer patients, GATA3 could not be considered an RFS prognosis marker for ER-positive patients (patients = 3951, HR = 0.94, 95% CI: 0.8–1.11, p-value = 0.46). Our results also demonstrated that an increased GATA3 mRNA expression was not significantly associated with a longer RFS time in the luminal A subtype (patients = 1933, HR = 0.9, 95% CI: 0.76–1.07, p = 0.23), luminal B subtype (patients = 1149, HR = 1.14, 95% CI: 0.94–1.38 p = 0.19) and basal subtype (patients = 618, HR = 0.85, 95% CI: 0.66–1.1, p = 0.22). Interestingly, the increased GATA3 expression level indicated a worse RFS survival outcome in the HER-2 group, although there was no significant difference (patients = 251, HR = 1.43, 95% CI: 0.97–2.1, p = 0.66). In particular, the subanalysis also revealed that an elevated GATA3 mRNA expression was related to a longer RFS time in the PR-positive patients (patients = 589, HR = 0.67, 95% CI: 0.47–0.95, p = 0.025, Fig. 6C) and the w/o lymph node metastasis group (Fig. 6H,I).

### IHC expression pattern utilities for clinical outcome prediction

**Figure 7:**
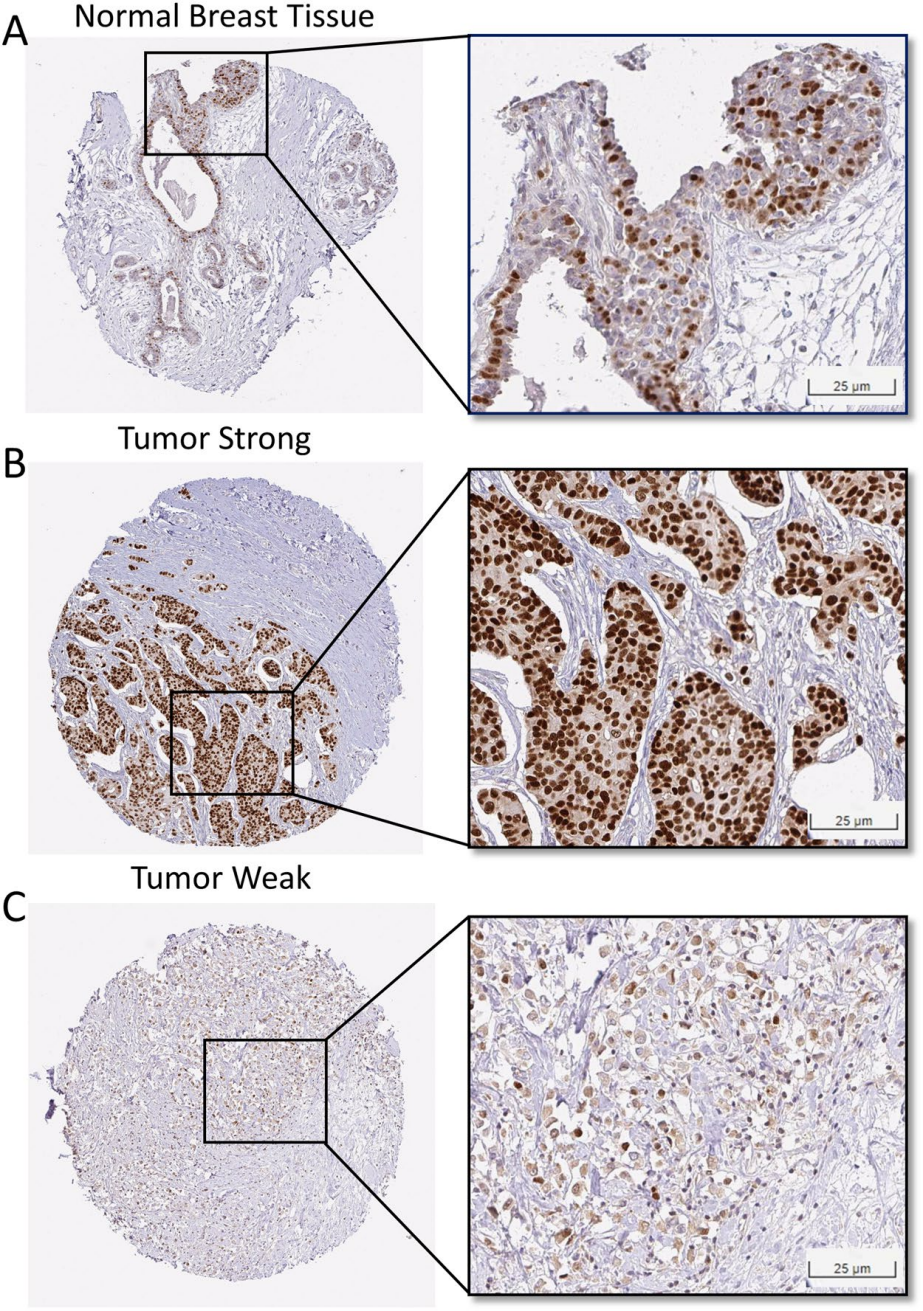
The IHC expression pattern of GATA3 in breast cancer. (**A**) Normal breast tissue. (**B**) Represented IHC of a high expression of GATA3. (**C**) Represented IHC of a low expression of GATA3.

To validate the potential application of GATA3 in the clinic, we extracted the characterized IHC images from the Human Protein Atlas. Normal breast tissue staining of GATA3 showed uneven positive staining in the nucleus with a weak–strong mixed pattern (Fig. 7A). However, in breast cancer tissue, there were distinguishable patterns between the strong and weak expression in the nucleus (Fig. 7B,C).

### Identification of the differentially expressed genes (DEGs) in GSE24249

**Figure 8:**
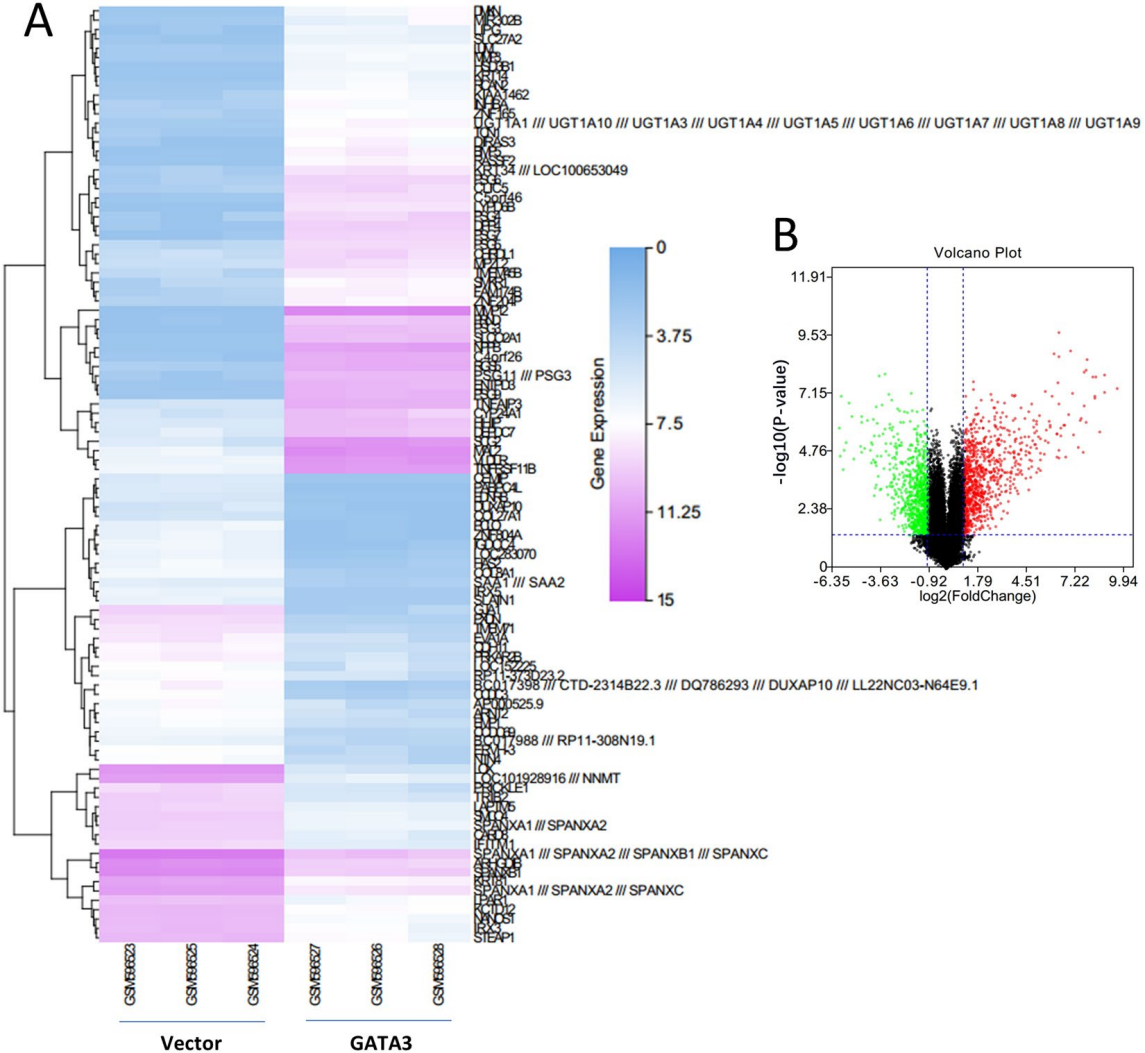
The DEGs of overexpressed GATA3 in the volcano plot and heatmap. (**A**) Heatmap of vector and GATA3 overexpression samples based on the identified 100 robust differentially expressed (50 top upregulated and 50 top downregulated) genes. The highest expression values of DEGs are displayed in pink, and the lower expression values gradually fade toward white color. the lowest expression values of DEGs are shown in blue, with higher values gradually fading toward white color. (**B**) The volcano plot shows the DEG distributions in both up- and downregulated genes. The green color shows the downregulated genes, while the red indicates the upregulated genes.

R software with the limma package was applied to screen DEGs from the gene expression dataset GSE24249 between control vectors and overexpressed GATA3 genes in MDA-MB231 breast cancer cells. A total of 600 DEGs were identified from this dataset, 240 upregulated genes and 360 downregulated genes (Fig. 8B). The top 50 up/downregulated genes were plotted in a heatmap (Fig. 8A).

### Biological classification and KEGG pathway enrichment analysis of DEGs

**Figure 9:**
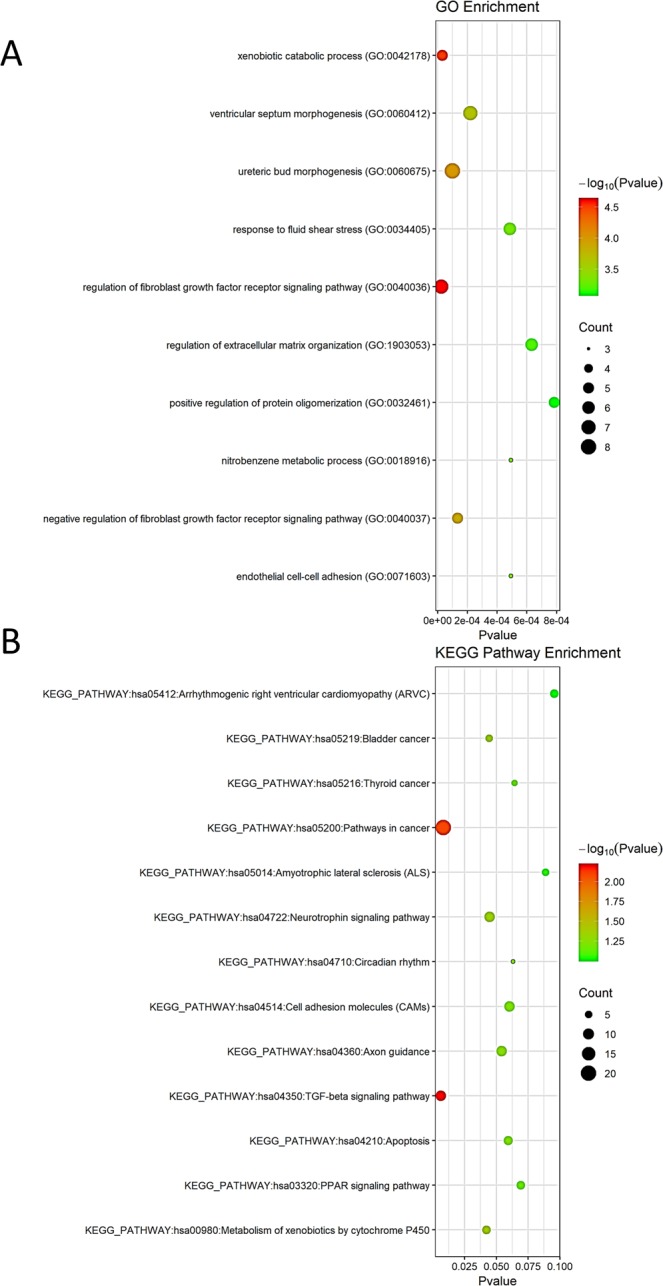
Functional enrichment analysis of Gene Ontology terms and KEGG biological pathway enrichment analysis of DEGs from GSE24294 (GATA3 overexpression). GO analysis according to the biological process. (**A**) Enrichment for GO ‘Biological Process’ terms of genes detected. The y-axis displays the fraction relative to all GO Biological Process terms. (**B**) Enrichment for KEGG. The figure shows terms on the x-axis that are significantly enriched (p-value).

To identify the DEG functions, all DEGs were analyzed in the DAVID database. The top 10 significant biological process GO terms enriched by the regulated genes were the following: nitrobenzene metabolic process (GO:0018916); endothelial cell-cell adhesion (GO:0071603); xenobiotic catabolic process (GO:0042178); negative regulation of fibroblast growth factor receptor signaling pathway (GO:0040037); regulation of fibroblast growth factor receptor signaling pathway (GO:0040036); positive regulation of protein oligomerization (GO:0032461); response to fluid shear stress (GO:0034405); ventricular septum morphogenesis (GO:0060412); regulation of extracellular matrix organization (GO:1903053); and ureteric bud morphogenesis (GO:0060675) (Fig. 9A).

For KEGG pathway enrichment analysis (Fig. 9B), the DEGs were found to be primarily enriched in the following top 10 KEGG pathways: hsa04350: TGF-beta signaling pathway; hsa05200: pathways in cancer; hsa00980: metabolism of xenobiotics by cytochrome P450; hsa05219: bladder cancer; hsa04722: neurotrophin signaling pathway; hsa04360: axon guidance; hsa04210: apoptosis; hsa04514: cell adhesion molecules (CAMs); hsa04710: circadian rhythm; hsa05216: thyroid cancer; hsa03320: PPAR signaling pathway; hsa05014: amyotrophic lateral sclerosis (ALS); and hsa05412: arrhythmogenic right ventricular cardiomyopathy (ARVC). These significantly enriched GO terms and KEGG pathways could help us deeply understand the function of DEGs overexpressing GATA3, which is involved in the occurrence and development of breast cancer.

### Gene set enrichment analysis (GSEA) of the GSE24249 dataset

**Figure 10:**
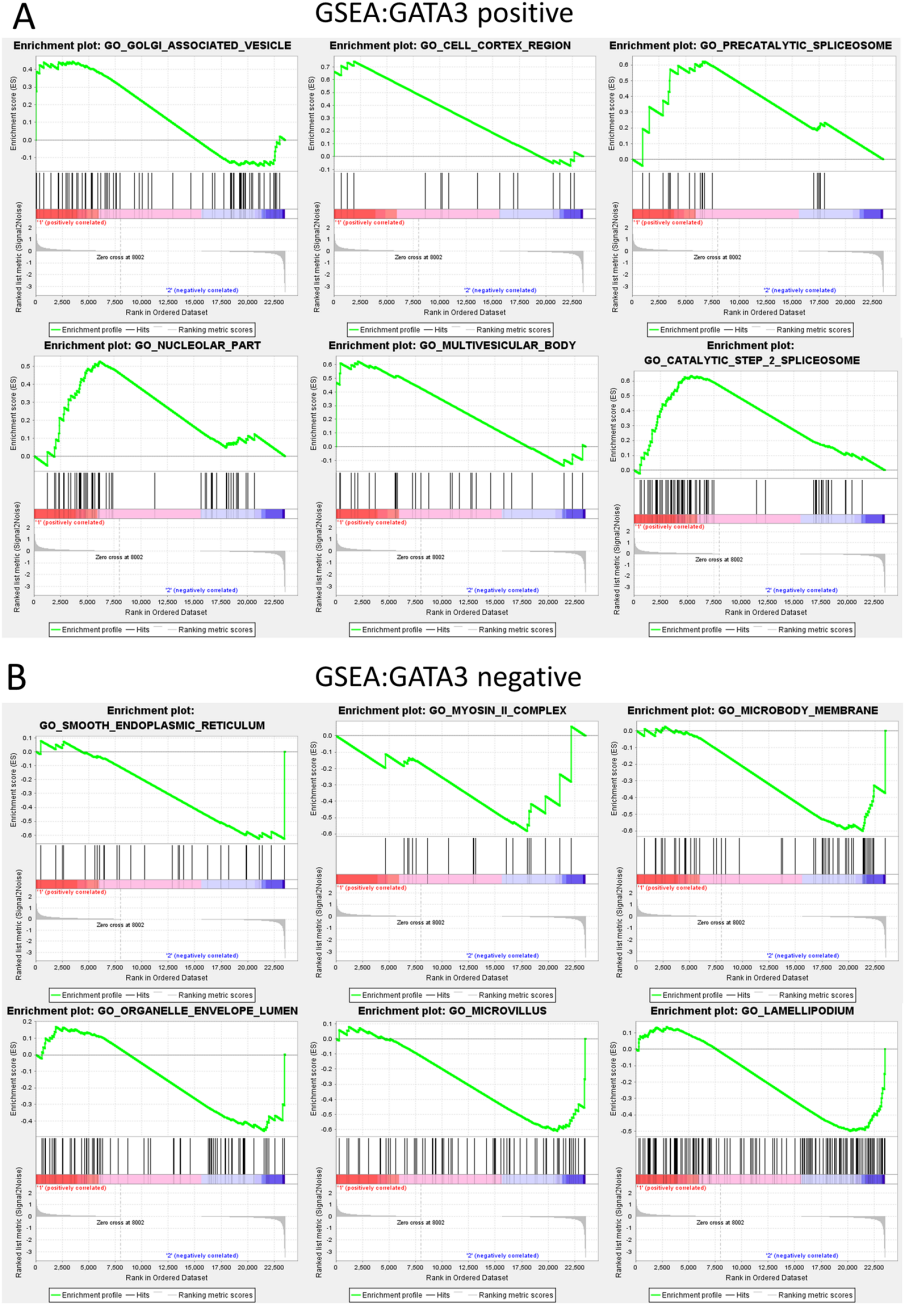
GSEA analysis of the top 6 GATA3-positive and -negative participating functions. (**A**) Top 6 terms of GSEA analysis result of GATA3 positive enriched functions by enrichment scores (ES). (**B**) Top 6 negative correlative functions.

Despite all DEGs, we further carried out GSEA analysis as GSEA considers experiments with genome-wide expression profiles from samples belonging to two classes (GATA3 vs Vector). The top six normalized enriched scores (NES) of GATA3 positive results were GO_GOLGI_ASSOCIATED_VESICLE_MEMBRANE, NES = 0.8148495; GO_CELL_CORTEX_REGION, NES = 1.36; GO_PRECATALYTIC_SPLICEOSOME, NES = 0.62; GO_NUCLEOLAR_PART, NES = 1.35; GO_MULTIVESICULAR_BODY, NES = 1.33; and GO_CATALYTIC_STEP_2_SPLICEOSOME, NES = 1.32 (Fig. 10A). In addition, the top six GATA3-negative GSEA results were GO_SMOOTH_ENDOPLASMIC_RETICULUM, NES = −1.46; GO_MYOSIN_II_COMPLEX, NES = −1.37; GO_MICROBODY_MEMBRANE, NES = −1.36; GO_ORGANELLE ENVELOPE_LUMEN, NES = −1.34; GO_MICROVILLUS, NES = −1.30; and GO_LAMELLIPODIUM, NES = −1.29 (Fig. 10B).

### Deciphering the complex network of protein-protein interactions (PPI)

**Figure 11:**
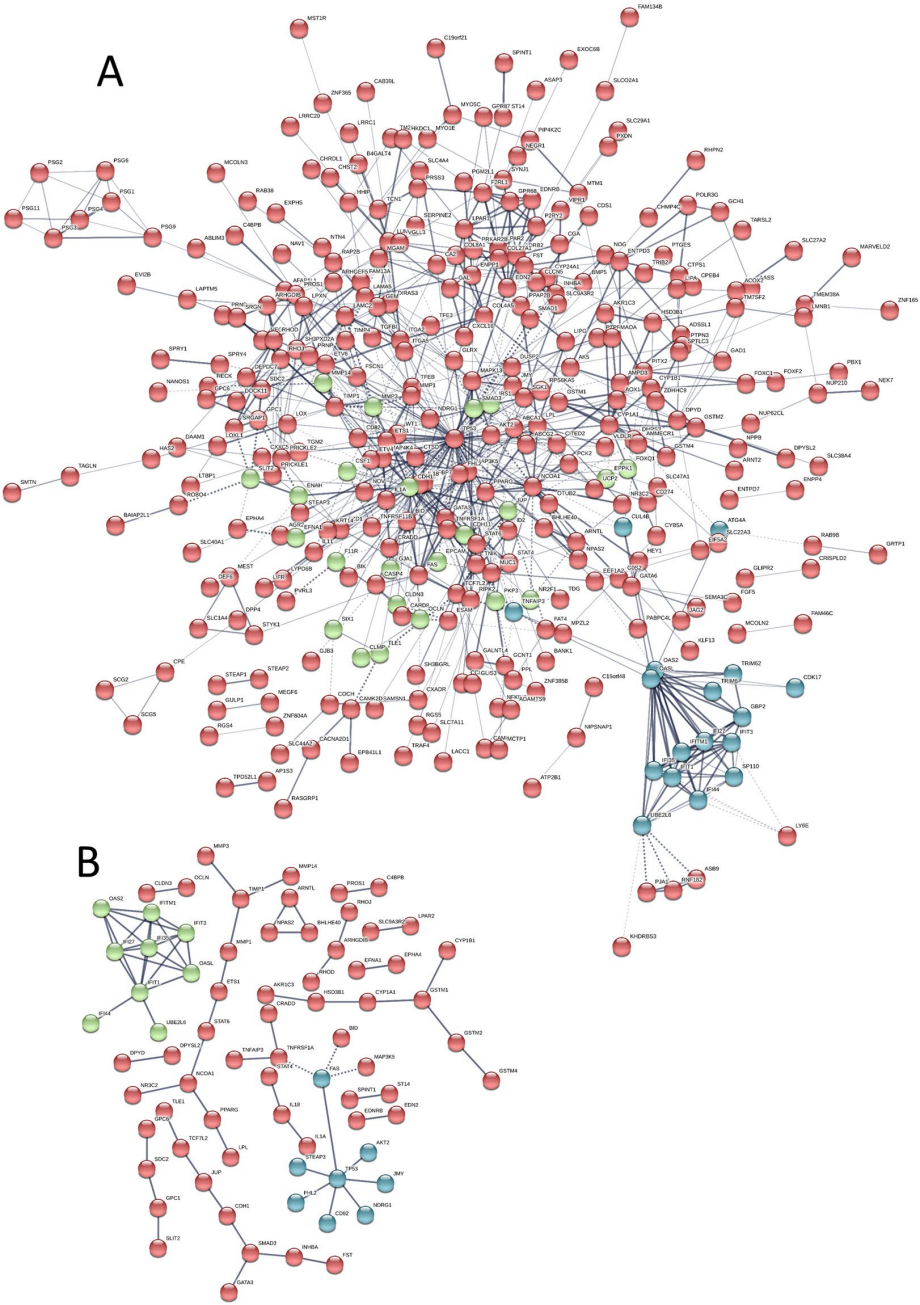
Protein-protein interaction network of DEGs. (**A**) Red, green and blue nodes represent the three clusters of all DEGs with connectivity >0.4; gray edges represent interactions between proteins. (**B**) Protein-protein interaction network of DEGs with connectivity >0.95. All disconnected nodes are hidden.

Protein-protein interactions help us explore molecular mechanisms. The interactions among the identified DEGs were analyzed by mapping with STRING with a combined score ≥0.4. The PPI network of the DEGs consisted of 531 nodes and 865 edges (Fig. 11A). Among these genes, TP53, SMAD3, CDH1, PPARG, and MAP3K5 showed the greatest degree in the PPI network (minimum required interaction score > 0.95), suggesting that GATA3 plays a key role in breast cancer malignancy transformation (Fig. 11B). The clusters by k-means show that OASL and OAS2 also participated in a small model involved in GATA3 functions. In addition, the following PPI functions were enriched: tissue development, FDR = 1.08e-07; regulation of locomotion, FDR = 1.72e-07; epithelium development, FDR = 1.72e-07; regulation of cell motility, FDR = 1.72e-07; regulation of cell migration, FDR = 1.84e-07; transcriptional misregulation in cancer, FDR = 0.00656; TNF signaling pathway, FDR = 0.0129; and pathways in cancer, FDR = 0.0242. These findings show that GATA3 is mainly involved in the development of breast cancer.

## Discussion

In recent decades, scientists have made tremendous improvements in understanding breast cancer. The prognosis markers are also widely explored. Among the known indicators, the key molecular signatures were well characterized as ER, PR and HER-2^27^. Based on these genes, breast cancer is classified into luminal A-, luminal B-, basal-like/triple negative- and Her-2-enriched subtypes^28^. Novel targets and signal pathways are continuing to emerge, such as Ezh2^29^, ADAM8^30^ and HMGA1^31^. These findings significantly contribute to the pathogenesis and development of breast cancer. However, clinical validations are further required.

GATA family members are key modulatory proteins known to be transcription factors in controlling several pathways. Nevertheless, these GATA transcription factor-related pathways have not yet been fully elucidated. As an important transcription factor family, they are an ideal and attractive module for investigating novel therapies for breast cancer^32^. However, basic explorations have shown significant contradictions in the specific roles of different GATA family members in breast cancer biology.

In our present study, by integrating analyses through GEPIA, Oncomine, TCGA, bcGenExMiner, Kaplan-Meier plotter, and the Human Protein Atlas, we systemically depicted the expression profiles of each GATA family member in breast cancer, revealing that the GATA family has a remarkable mRNA expression difference between cancer and normal breast tissue. These results demonstrated that the GATA family played important roles in breast cancer development.

Thus far, GATA1 is believed to participate in acute megakaryocytic leukemia and Down syndrome^33^. Li Yan *et al*. reported that GATA1 could induce the EMT process by binding to the *E-cadherin* promoter, downregulating E-cadherin expression through PAK5 oncogenic signaling in breast cancer. GATA1 is also a promoter binder factor of peroxiredoxin 5 in human breast cancer cells through inhibiting apoptosis^34^. There is also a report that GATA1 was an independent poor prognosis marker in breast cancer^35^. The low expression of GATA1 and GATA2 is associated with the aggressiveness and poor outcome of clear cell renal cell carcinoma^36^. Additionally, GATA2 is identified as a poor prognosis marker in colorectal cancer, prostate cancer and hepatocellular cancer^37–39^. In breast cancer, GATA2 was found to be a key epigenetic regulator for G9a, which impacts breast cancer cell survival and tumorigenesis^40^. However, there is no report on the role of GATA2 in prognosis in breast cancer. Contractional reports on GATA3 for breast cancer are still available. An early report in 2008 with 3,119 cases showed that GATA3 was highly associated with ER expression but neither had an independent prognostic value nor was useful as a prediction marker for the effect of tamoxifen in ER-positive patients^41^. However, other studies demonstrated that GATA3 was an independent favorable prognosticator^42,43^. There was also a report that showed that high GATA3 expression was associated with an unfavorable prognosis in breast cancer patients^7^. Inconsistent investigations on the protective effect of GATA3 were also reported^44–46^. The potential explanation for this phenomenon may be that GATA3 acts differently in various breast cancer subtypes. Further investigations on the GATA3 mechanism are still warranted. Kiyoshi *et al*. reported that the status of GATA4, but not other GATA family members, including GATA3, was an independent prognostic factor for the disease-free and breast cancer-specific survival of invasive ductal carcinoma patients^47^. However, our results are not consistent with their conclusion. ERBB2 is a key regulator in breast cancer, and the strong negative feedback association between GATA4 and ERBB2 may contribute to the transcriptional dysregulation of ERBB2 gene expression in breast cancer^48^. The promoter hypermethylation of GATA5 is believed to participate in invasive breast cancer development^49^. Interestingly, the dual role of GATA5 in the activation of the progesterone receptor gene promoter contributes to the susceptibility of breast cancer^50^. However, GATA5 has not been widely investigated in breast cancer. GATA6 is reported to be overexpressed in breast cancer, consistent with our pan-cancer analysis. The epithelial-mesenchymal transition (EMT) process was under the regulation of GATA6 via upregulating Slug expression. However, there is only one report showing that GATA6 overexpression could be considered an independent prognostic marker with a favorable outcome in breast cancer^51^. Our research result for GATA6 is inconsistent with their report. Interestingly, in digestive system cancers, such as esophageal adenocarcinoma, pancreatic cancer, colorectal cancer or cholangiocarcinoma, GATA6 could be used as a prognostic tumor marker for different mechanisms^52–55^. These research findings reveal the important role of GATA6 in digestive system malignancy progression.

To clarify our research focus, we summaries our study approaches and workflow in Fig. S2. Our findings from the integrative databases and bioinformatics analysis of this study suggest that GATA3, but not the other GATA family members, might be a potential prognostic biomarker and target for new therapies for breast cancer. However, the different expressions of the GATA family members in pan-cancer analysis reveal that the GATA family plays an important role in malignancy transformation. In addition, GATA3 may mainly interact with TP53, SMAD3, and CDH1 genes to regulate endothelial cell-cell adhesion by the FGF and TGF pathways. However, more investigations need to be applied to fully reveal the role of GATA3 in breast cancer for further translational study.

## Supplementary information

Supplementary figures with legends
