# Supplementary material for: Comprehensive analysis of the GATA transcription factor gene family in breast carcinoma using gene microarrays, online databases and integrated bioinformatics
Source: Sci Rep. 2019 Mar 14;9:4467. doi: 10.1038/s41598-019-40811-3 (PMC6418253; doi:10.1038/s41598-019-40811-3)
Supplement: Supplementary file 1 — Supplementary figures with legends [file 41598_2019_40811_MOESM1_ESM.docx]

**Comprehensive analysis of the GATA transcription factor gene family in breast carcinoma using gene microarrays, online databases and integrated bioinformatics**

Shan Yu^1^, Xuepeng Jiang^2^, Juan Li^1^, Chao Li^3^, Mian Guo^4^, Fei Ye^5^, Maomao Zhang^6^, Yufei Jiao^1^, Baoliang Guo^7^*

^1^Department of Pathology, the Second Affiliated Hospital of Harbin Medical University, Harbin 150001, China

^2^Department of General Surgery, the Heilongjiang Power Hospital, Harbin, 150090, China

^3^Department of Orthopedics, the Second Affiliated Hospital of Harbin Medical University, Harbin 150001, China

^4^Department of Neurosurgery, the Second Affiliated Hospital of Harbin Medical University, Harbin 150001, China

^5^Department of Pathology, Harbin Medical University, Harbin 150001, China

^6^The Key Laboratory of Myocardial Ischemia, Department of Cardiology, the Second Affiliated Hospital of Harbin Medical University, Harbin 150001, China

^7^Department of General Surgery, the Second Affiliated Hospital of Harbin Medical University, Harbin 150001, China

Correspondence to Baoliang Guo, Department of General Surgery, the Second Affiliated Hospital of Harbin Medical University, 246 Xuefu Street, Nangang District, Harbin, 150001, China. Email: guobaoliang9999@163.com


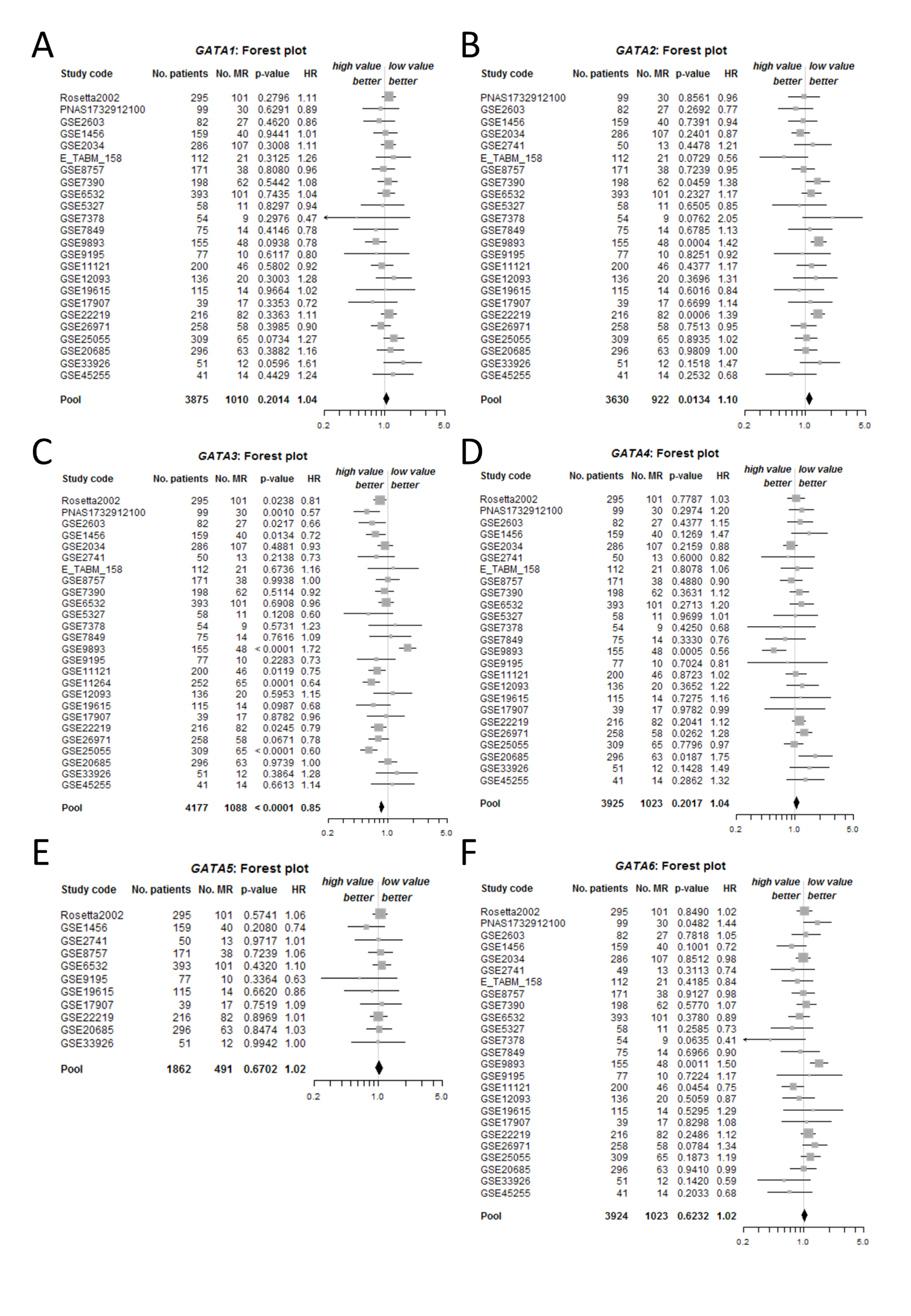
Figure S1: Forest plot of GATA family members on pooled survival data by GEO and other datasets.


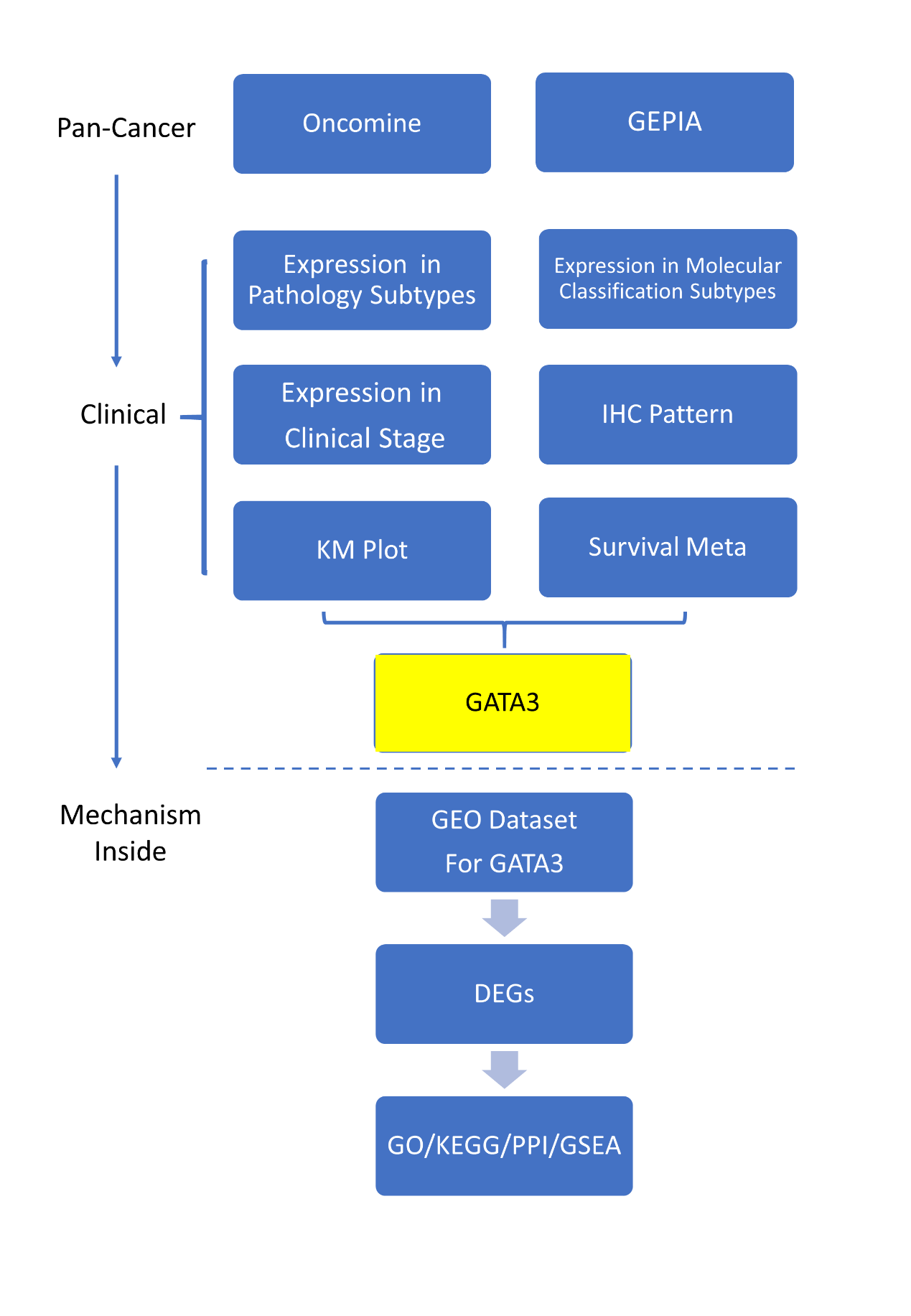
Figure S2: The flow chart of our study design.
